# Drinking Strategies: Planned Drinking Versus Drinking to Thirst

**DOI:** 10.1007/s40279-017-0844-6

**Published:** 2018-01-24

**Authors:** Robert W. Kenefick

**Affiliations:** Thermal and Mountain Medicine Division, US Army Research Institute, Natick, MA USA

## Abstract

In humans, thirst tends to be alleviated before complete rehydration is achieved. When sweating rates are high and ad libitum fluid consumption is not sufficient to replace sweat losses, a cumulative loss in body water results. Body mass losses of 2% or greater take time to accumulate. Dehydration of ≥  2% body mass is associated with impaired thermoregulatory function, elevated cardiovascular strain and, in many conditions (e.g., warmer, longer, more intense), impaired aerobic exercise performance. Circumstances where planned drinking is optimal include longer duration activities of >  90 min, particularly in the heat; higher-intensity exercise with high sweat rates; exercise where performance is a concern; and when carbohydrate intake of 1 g/min is desired. Individuals with high sweat rates and/or those concerned with exercise performance should determine sweat rates under conditions (exercise intensity, pace) and environments similar to that anticipated when competing and tailor drinking to prevent body mass losses >  2%. Circumstances where drinking to thirst may be sufficient include short duration exercise of <  1 h to 90 min; exercise in cooler conditions; and lower-intensity exercise. It is recommended to never drink so much that weight is gained.

## Introduction

The two most common schools of thought regarding the best fluid intake practices during exercise are programmed drinking versus drinking to thirst or ad libitum drinking [1, 2]. These fluid consumption practices have been a topic of recent debate in the literature [3, 4]. Consensus statements and sports medicine society position stands either focus on maintaining performance and reducing cardiovascular and thermoregulatory strain, in the case of the American College of Sports Medicine guidelines [5], or preventing hyponatremia, in the case of the Statement of the Third International Exercise-Associated Hyponatremia Consensus Development Conference [6]. Differences in emphasis have resulted in recommendations for fluid intake strategies that may appear to be at odds, with one position stand recommending programmed drinking [5] while a consensus statement [6] recommends an ad libitum*/*drink to thirst strategy. Despite apparent differences, both strategies seek to prevent over/under hydration and preserve performance. However, the success of either strategy will depend on the context of the event (duration, intensity, and environment), the characteristics of the individual (fitness, acclimatization status, etc.), and the goals of the individual exercising, training, or competing.

## Definitions and Objectives of ‘Programmed Drinking’ and ‘Drink to Thirst’

Defining the terminology of each fluid intake strategy is important to avoid confusion and so that specific differences between the two strategies can be fully understood. For the purposes of this review, the operational definitions provided in Sects. 2.1 and 2.2 are used.

### Programmed Drinking: The Use of a Pre-Established Drinking Plan

While drinking to thirst could be included in the definition of programmed drinking, typically this term refers to drinking predetermined amounts of fluid with the purpose of minimizing fluid losses. This fluid intake strategy is based on the fact that there is considerable variability in sweating rates and sweat electrolyte concentrations between individuals, thus requiring a customized fluid replacement program. The objective of programmed drinking is to prevent dehydration and over-drinking (±  2% body mass) by drinking to approximate sweat losses, with the goal of attenuating potential exercise performance impairment, reducing cardiovascular and thermoregulatory strain associated with dehydration, decreasing the risk of heat illness (heat exhaustion, heat stroke), and preventing hyponatremia [5]. Determination of sweat rate can be accomplished by measuring acute changes in body weight before and immediately after exercise. In the absence of drinking, change in body weight can be used as an approximation of the volume of sweat lost (e.g., 1 kg = 1 L); however, there may be some small sources of error in this assumption.

### Drinking to Thirst: The Use of the Sensation of Thirst as the Only Stimulus to Drink

For the most part, ‘drink to thirst’ has been used inter-changeably with ‘ad libitum drinking’ [7]. ‘Ad libitum drinking’ is defined as the consumption of fluid whenever, and in whatever volume, desired [8, 9]. A recent study investigating the differences between ‘drinking to thirst’ and ‘ad libitum’ drinking reported that when volunteers were instructed to use either strategy, the physiologic and perceptual outcomes were similar [10]. For the purposes of this review, the use of ‘ad libitum’ drinking in the literature is taken to mean ‘drinking to thirst’ and these terms are used synonymously. The objective of ‘drinking to thirst’ is to use the innate thirst mechanism to guide fluid consumption with the goal of preventing the development of exercise-associated hyponatremia (EAH) and excessive dehydration [6].

## Fluid Balance and Thirst

**Fig. 1:**
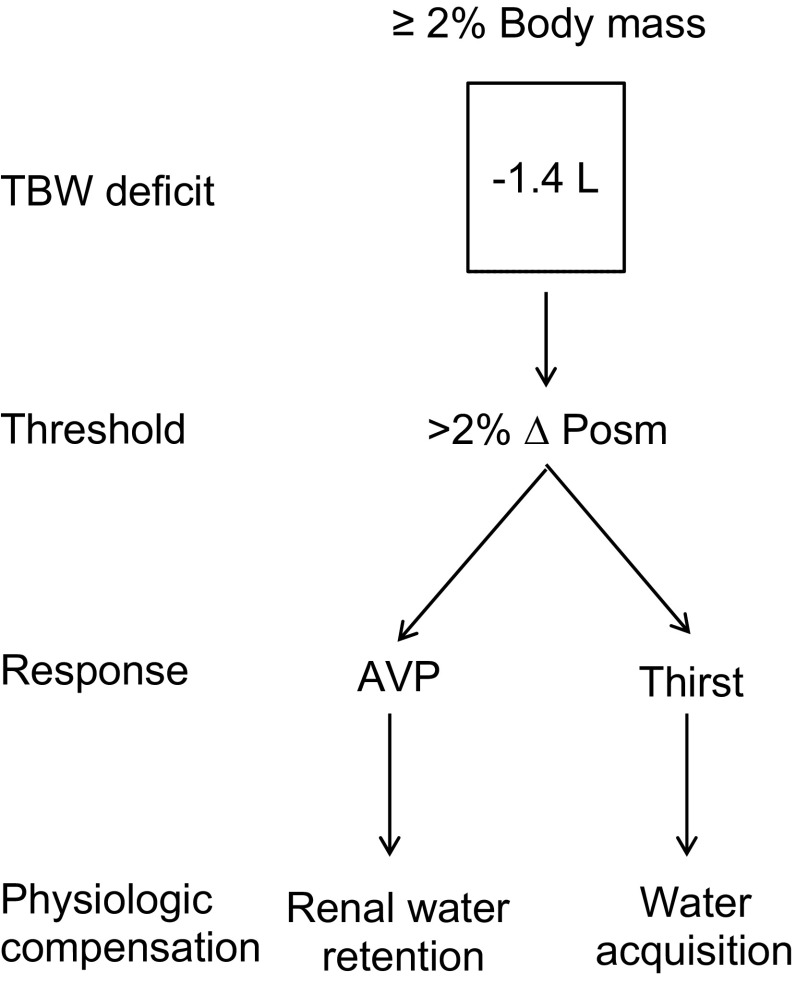
Regulation of body water balance in response to body water deficit typical of exercise/fluid restriction for a 70 kg individual. Schematic includes the estimated magnitude of dehydration (2% body mass loss) required to stimulate the osmotic-dependent response for compensatory water conservation and acquisition (thirst). A change in total body water is equated with a change in body mass (1 L = 1 kg), whereby dehydration is then expressed as a percentage of body mass in accordance with: (Δ body mass/initial body mass) × 100 or, for this example, (1.4 kg/70 kg) × 100 = 2%. *AVP* arginine vasopressin, *Posm* plasma osmolality, *TBW* total body water

Net body water balance (loss = gain) is regulated remarkably well day-to-day as a result of thirst and hunger drives coupled with ad libitum access to food and beverages to off-set water losses [11]. However, when there is a mismatch where fluid intake is less than fluid loss, dehydration results. Dehydration is defined as a body water deficit greater than normal daily fluctuation [12] or when body water deficits exceed 2 standard deviations in normal body mass variability (≥  2% of body mass) [13, 14]. When at rest, this level of dehydration also represents an approximate threshold where compensatory fluid regulatory actions and stimulus for fluid acquisition occur (≥  2% body mass) [15, 16]. These compensatory actions are triggered principally by an elevation in plasma osmolality (Posm) and, to lesser degree, a reduction in plasma volume [12, 17]. During exercise, particularly in the heat, plasma volume decreases because it provides the fluid for sweat, and as a result, Posm increases because sweat is hypotonic (sodium poor) relative to plasma. An ~  2% increase in Posm (~  6 mmol/kg) is commonly referenced as an osmotic threshold for compensatory renal water conservation and water acquisition (thirst), which is approximately the equivalent of ≥  2% loss of body mass (1.4 L at 70 kg; Fig. 1) [12]. The sensitivity of osmoreceptors in regulating anti-diuretic hormone release and stimulating thirst is enhanced by relatively small losses of volume. However, volume-mediated thirst requires a much larger loss (~  10% blood volume) and plasma volume losses are only ~  0.14 L with a loss of ~  2% body mass [18].

**Fig. 2:**
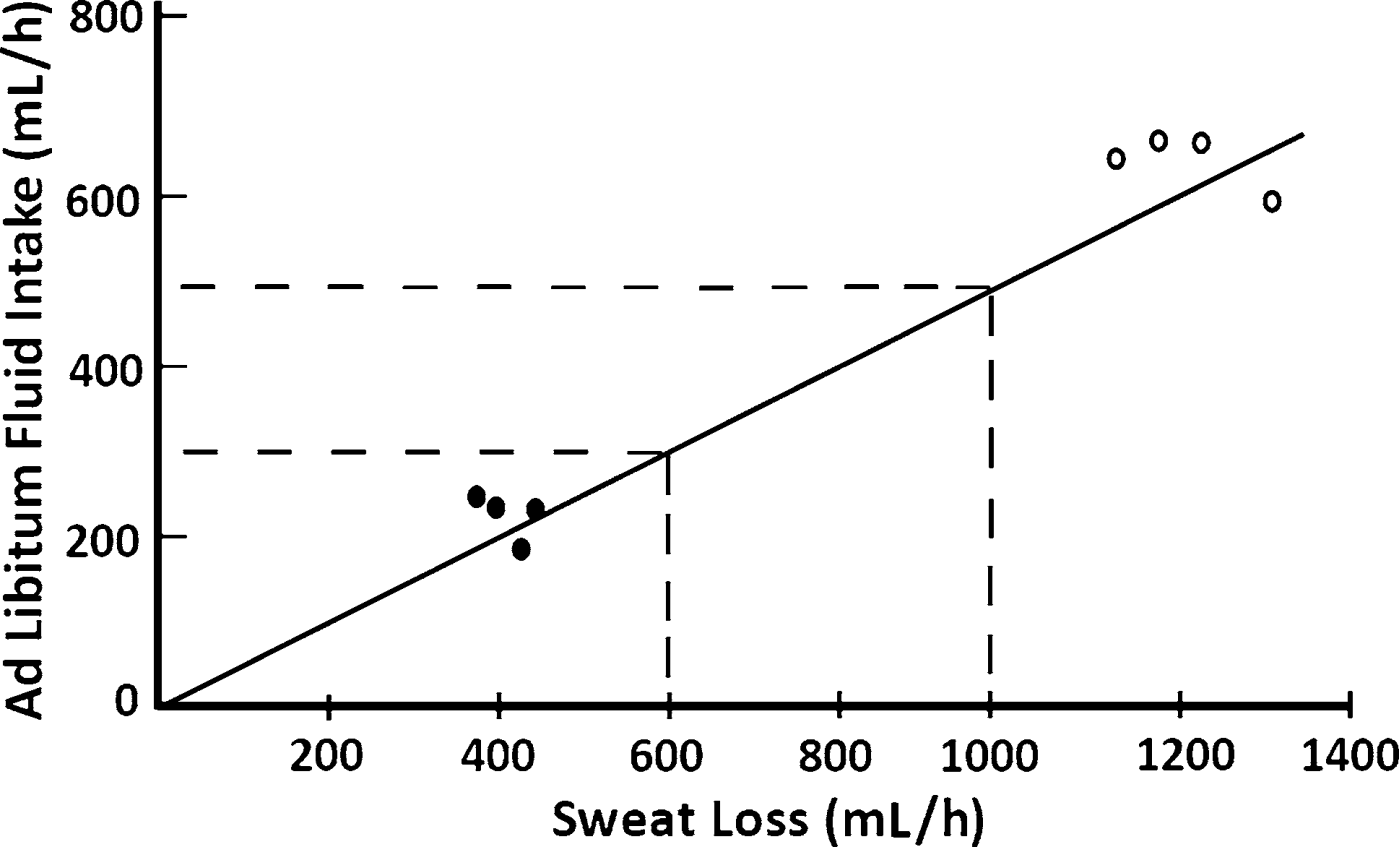
Ad libitum fluid intake vs. sweat losses during treadmill walking in cool (24 °C; filled circle) and hot (49 °C; open circle) environments. Ad libitum fluid intake equals ~  50% of fluid losses (adapted from Greenleaf and Sargent [19] with permission)

**Fig. 3:**
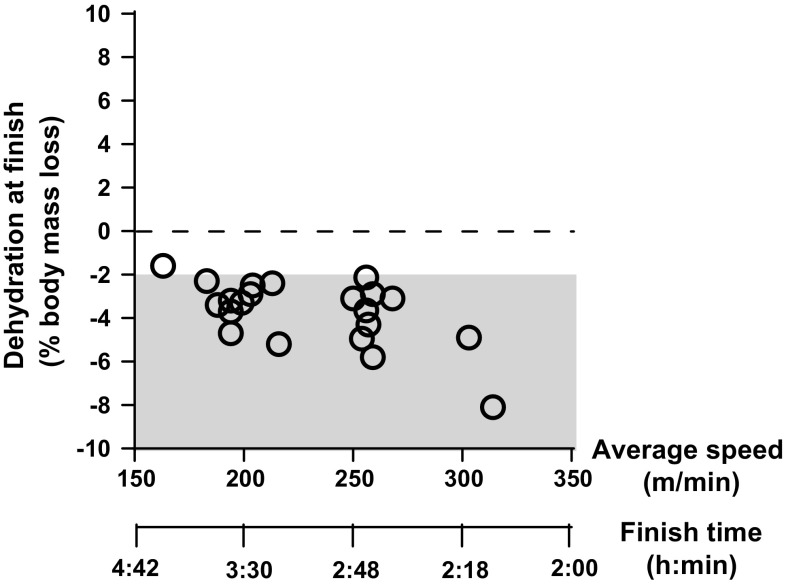
Level of post-race dehydration vs. average running speed and finishing time for 42 km when drinking ad libitum. Adapted from Cheuvront et al. [32]

While sensation of thirst works well at rest [19], it is less sensitive during exercise. Observations of the lack of sensitivity of thirst in the maintenance of total body water during exercise have been reported in the literature over many years. Dill et al. [20] observed that when a man and a dog walked 32 km in a hot environment, the dog maintained its weight balance while the man lost about 3 kg of his body mass despite the fact that water was available ad libitum to both. Dill et al. [20] concluded that during exercise, man undergoes a decrease in body mass when water is drunk ad libitum. During periods of high sweat rates (>  1.0 L/h) humans practicing ad libitum drinking have been reported to markedly under-consume fluid [13, 18, 21–23]. Greenleaf et al. [19] reported that when drinking ad libitum, subjects consumed approximately half of the fluids lost during exercise in cool and hot environments (Fig. 2). Even when drinking ad libitum, subjects performing a half-marathon reported feeling more thirsty than subjects adhering to programmed drinking in trials [24]. Thirst is also alleviated before complete rehydration is achieved [25] as oropharyngeal cues trigger thirst satiation before volume is fully restored [26–31]. Greenleaf et al. [19] further reported that following experimental trials, subjects reported feeling fully recovered and were not thirsty despite having a water deficit of 4–5 L (Fig. 2). In a further example, Cheuvront et al. [32] examined group means from 14 marathon studies conducted in a range of environments (10–28 °C) with runners of wide ranging abilities (2 h 10 min to 4 h; Fig. 3) and concluded that ad libitum drinking commonly led to excessive dehydration (>  2% body mass loss).

It should also be appreciated that the mechanisms that stimulate the sensation of thirst are subject to numerous influences [33] and sensitivity to these signals during exercise is likely different given the physiological state during exercise (elevated heart rate and respiration; decrease in renal blood flow and plasma volume; elevation in anti-diuretic and other fluid regulatory hormones, etc.) compared to rest. Further, it should be recognized that when dealing with exercising children or elderly individuals, the sensation of thirst has been reported to be less sensitive for both populations [34].

## Dehydration: Physiological Responses and Exercise Performance

The majority of the dehydration/exercise performance literature suggests that during exercise, dehydration increases physiological strain as measured by elevations in core temperature, heart rate, and perceived exertion responses [35]. Also, the greater the body water deficit, the greater the increase in physiological strain [21, 36–38]. As discussed in Sect. 3, when dehydration occurs due to sweat loss, a state of hyperosmotic hypovolemia results and increases proportionally to the decrease in total body water [11]. The resulting hyperosmolality can delay thermoregulatory cutaneous vasodilation and sweating, increasing thresholds for both [39, 40]. As a result, dehydration reduces the sweating rate for any given body core temperature, decreases evaporative heat loss [38], and increases heat storage [39, 41]. Due to a reduction in circulating plasma volume, heart rate increases secondary to a reduction in stroke volume [42, 43]. Heat stress in combination with dehydration further exacerbates these cardiovascular responses because it creates competition between the central and peripheral circulation for limited blood volume [44], which further magnifies the physiologic strain for a given exercise task [36–38].

**Fig. 4:**
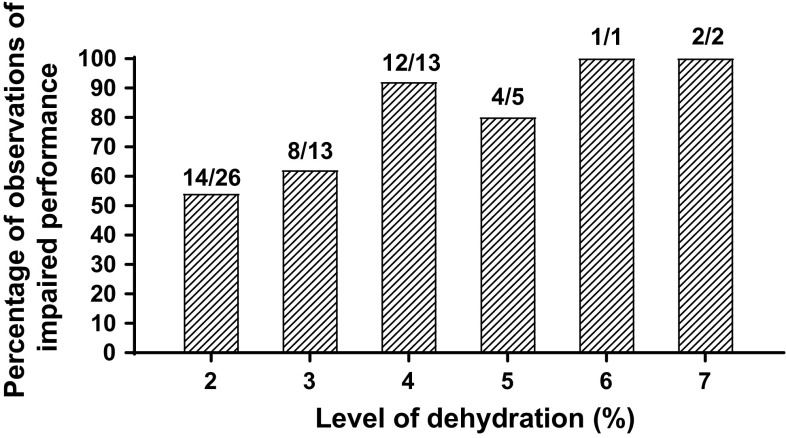
Review of dehydration effects on performance in 34 endurance exercise/dehydration studies. Fractions above bars represent the number of statistically significant (*p* < 0.05) observations (numerator) of total observations (denominator) at the specified level of dehydration. 41 of 60 total observations (68%) were significantly (*p* < 0.05) impaired by dehydration ≥  2% body mass. Adapted from Cheuvront and Kenefick [12])

In regards to exercise performance, there is an overall consensus in the literature that dehydration of ≥  2% body mass represents a threshold at which aerobic exercise performance or endurance becomes impaired [3, 5, 12, 45]. We previously evaluated 34 endurance exercise/dehydration studies, which included 60 separate observations (Fig. 4) [12]. A total of 41 of 60 observations (68%) were significantly (*p* < 0.05) impaired by dehydration ≥  2% body mass. Independent of *p* value (*p* < 0.05), the number of studies reporting a reduction in performance for endurance exercise of ≥  2% body mass loss was 53 of 60 negative observations or 88%. For more detail regarding the impact of dehydration on physiological responses and exercise performance, more comprehensive reviews are recommended [11, 12, 46, 47].

Many of the studies reviewed were conducted in a laboratory, which can be considered to be a limitation, as laboratory conditions are less ecologically valid by design. Valid criticisms include achievement of dehydration before (rather than during) exercise and unrealistically low air flow rates. However, a review of dehydration studies where water loss occurred during exercise had similar conclusions [46]. Furthermore, in one of the better examples of a field-valid study of endurance sport, Casa et al. [48] examined the impact of dehydration (~  2% body mass loss) on trail running performance. Run times were ~  5% slower when completing the race while dehydrated.

**Fig. 5:**
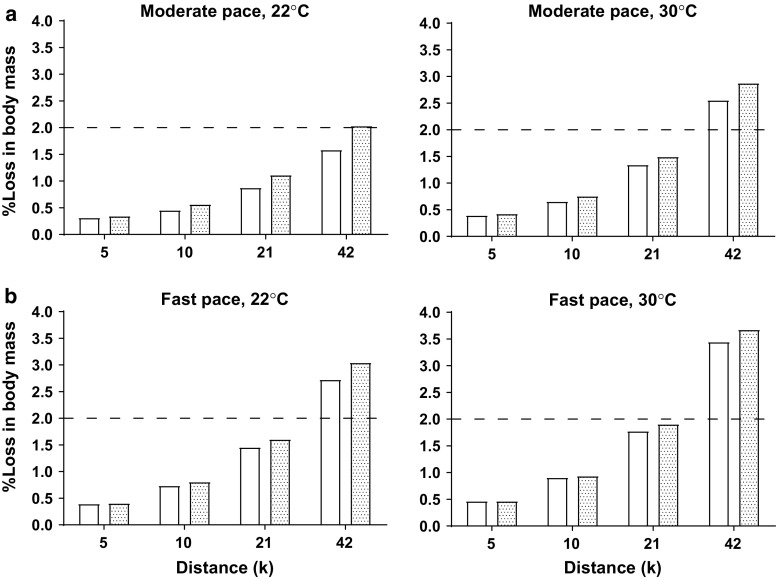
Percentage loss in body mass predicted from sweat rate for 60 and 80 kg runners of average ability **a** during 5 km (25 min), 10 km (60 min), 21 km (130 min), and 42 km (270 min) and competitive ability **b** during 5 km (21 min), 10 km (43 min), 21 km (95 min), and 42 km (200 min) road races. The dotted line demarks 2% body mass loss. Losses assume no fluid intake. Adapted from Kenefick and Cheuvront [49]

It is important to note that when exercise commences in a well-hydrated state, accumulated fluid loss and the subsequent development of sensations of thirst can take time and will be dependent on numerous factors (e.g., environment, exercise intensity and duration, sweat rate). To bolster the point that dehydration requires time to accumulate, we predicted sweat losses for two hypothetical runners of small and larger body sizes over distances from 5 to 42 km (marathon) in temperate (22 °C) and warm conditions (30 °C) [49]. These predictions illustrated differences in fluid needs for differing exercise durations, intensities, environments, and body sizes. Fluid losses were expressed as the percentage loss in body mass relative to a threshold of 2% loss over the duration of each event (Fig. 5a, b). What could be observed was that for finishing times typical of the majority of runners, fluid losses are <  2% of body mass for distances up to 21 km and it is not until marathon distance in hot conditions (30 °C) that larger individuals (80 kg) lose >  2% of body mass by the very end of the event (Fig. 5a). For faster, more competitive runners (Fig. 5b), fluid losses are greater for both smaller and larger runners and exceed 2% body mass loss in both warm and hot conditions during the marathon but are below 2% body mass loss for the other distances (5–21 km). These modeled loss estimates are conservative, as the equations used are not specifically designed for sport or sport clothing. However, they do illustrate that fluid replacement becomes increasingly critical during higher-intensity and longer-duration exercise, particularly when temperatures are warmer.

The threshold of ±  2% body mass loss appears to be significant in regards to a number of factors, including fluid conservation, stimulation of thirst, and impairment of thermoregulatory and cardiovascular function and exercise performance. Thus, it stands to reason that during exercise, a fluid replacement strategy that maintains hydration state within ±  2% body mass would be successful in the preservation of physiological and exercise performance. As demonstrated by our fluid need predictions, fluid loss of 2% body mass takes time to accumulate and will be dependent on the environment, exercise intensity, and duration of the event.

## Ad Libitum Drinking and Exercise Performance

Ad libitum or drink to thirst studies involving endurance running [50] and half marathon [24] and marathon [51] events have reported greater cardiovascular and thermoregulatory strain [24] but no differences in plasma volume or osmolality [49], and no differences in running performance [24, 50, 51]. Ad libitum cycling studies have reported that cardiovascular responses [52], thermoregulation [52, 53], and performance [52, 53] are not different from programmed drinking. In contrast, Bardis et al. [54] recently compared ad libitum with prescribed drinking during a 30 km cycling performance in the heat and concluded that matching fluid intake with sweat losses provided a performance advantage due to lower thermoregulatory strain and greater sweating responses. Ultra-running studies examining ad libitum drinking have concluded that this strategy led to no incidences of hyponatremia [55] and did not impact performance despite body mass losses >  3% [56, 57] and conclude that drinking beyond thirst is not required to maintain hydration during ultra-endurance events. Where ultra-endurance exercise (activity consisting of many hours/days) is concerned, it is important to mention that these activities can result in significant non-fluid mass losses and non-water fluxes that make determination of body mass changes, and thus fluid losses, difficult to determine and interpret.

Overall, the findings of the ad libitum/drink to thirst literature support the idea that maintaining fluid balance within ±  2% body mass is dependent on the environment, exercise intensity, and duration of the event. Ad libitum/drink to thirst studies have been conducted in low ambient temperatures [50, 55], during events of 2 h or less [24, 50, 52, 53], and in ultra-events that are longer in duration and tend to have lower exercise intensities [55–57], they tend to have lower exercise intensities. Many of the ad libitum/drink to thirst studies have been performed in field settings or during competition (vs. laboratory) where there is greater air flow, greater convective heat loss and, as a result, reduced cardiovascular and thermoregulatory strain. Also, in the majority of field studies or competitions, volunteers started exercise in a euhydrated state and progressively dehydrated during the event or trial. Thus, ≥ 2% body mass loss may not be achieved until the end of the event, or not at all in the case of shorter events/trials.

## Conclusions

Given predicted fluid requirements for differing exercise durations, intensities, environments, and body sizes, it would appear that conditions exist where ad libitum/drink to thirst fluid intake *will* be sufficient to meet needs, i.e., maintenance of fluid balance within ±  2% body mass. For individuals who are less concerned with performance or performing activities at lower intensities, particularly in cooler weather, a fluid replacement plan may not be as important because fluid losses may not approach 2% body mass loss. These conditions include activities or competition of < 1–2 h of duration, that are of lower exercise intensity, and that take place in cool or temperate environments.

However, there are also conditions where programmed drinking is necessary to meet requirements and a tailored programed drinking strategy will need to be employed to avoid potential thermoregulatory, cardiovascular, and exercise performance impairment (2% body mass loss). These conditions include activities or competition that are longer in duration (> 90 min to 2 h), are of higher exercise intensity, take place in warm or hot environments, or for which fuel intake at a particular rate is desired (e.g. 1 g carbohydrate/min). Thus, a programmed drinking strategy should be tailored to prevent body mass losses or gains of ±  2% body mass during these activities [5].

As the practice of ad libitum/drink to thirst fluid intake appears to result in fluid replacement of about half of fluid losses [18], this strategy would appear to be successful in the prevention of hyponatremia. However, humans consume fluids for reasons outside of thirst/fluid replacement and, while rare, cases have been documented where individuals have consumed fluids ‘according to thirst’ but over-drank and became hyponatremic [6]. When consuming fluid ad libitum/to thirst, or if consuming fluid according to a predetermined program, it is important to *never* consume so much fluid that weight is gained.
